# Children with autism in the Greek educational system: ongoing challenges during the COVID-19 pandemic

**DOI:** 10.1192/bji.2023.5

**Published:** 2023-08

**Authors:** Sotiria Mitroulaki, Maria Samakouri, Aspasia Serdari

**Affiliations:** 1 Special Educator, Department of Child and Adolescent Psychiatry, Medical School, Democritus University of Thrace, Dragana, Alexandroupolis, Greece. Email: sotiria6@hotmail.com; 2 Professor of Psychiatry, Department of Psychiatry, Medical School, Democritus University of Thrace, Dragana, Alexandroupolis, Greece; 3 Associate Professor of Child and Adolescent Psychiatry, Department of Child and Adolescent Psychiatry, Medical School, Democritus University of Thrace, Dragana, Alexandroupolis, Greece

**Keywords:** Inclusion, COVID-19, autism spectrum disorder, education, policy

## Abstract

The inclusion of children with autism spectrum disorder (ASD) in typical educational settings has only recently gained momentum in Greece, responding to the recommendations of the international conventions. Reform of special education legislation spotlights the inclusion of children with autism in mainstream schools. The principal goal is to accept the diversity and heterogeneity of all students. This paper presents the educational policy for children with ASD in Greece and comments on teachers’ perceptions of inclusion. School closures due to the COVID-19 pandemic had an adverse impact on children's lives and created a new environment with different demands for educational inclusion.

In recent decades, there has been an attempt globally to reform mainstream schooling based on a value system that respects and welcomes heterogeneity of all children, including those with autism spectrum disorder (ASD). The prevalence rate of ASD in 10- and 11-year-old children in Greece has been estimated at 1.15%.^1^ The shift to inclusion as the ideal practice has led to the development of an educational system that takes into consideration all children's needs and above all celebrates diversity. Inclusion is defined as the process that aims to give all children and young people access to the whole range of educational and social opportunities within the school environment.^2^ However, even though children with autism should experience that inclusion, its degree and nature are often deeply questionable.^3^ This fact could be explained bearing in mind, on one hand, that children with milder symptoms and no marked developmental delay may go undiagnosed for years, struggling with no support,^4^ and on the other hand, that teachers may be hesitant to support inclusion because of their lack of experience. The aim of this article is describe the evolution of inclusive education for children with ASD in Greece through reviewing the relevant laws and ministerial decisions before and during the COVID-19 pandemic.

## Educational inclusion policy for children with ASD in Greece

Globally, over the past 50 years the education of children with special educational needs (SEN) and/or disabilities has changed drastically. There has been a shift from exclusion and segregation to integration and finally inclusion. The history of special education in Greece has its origins back at the beginning of the 20th century. It has evolved slowly over the years, as the state was reluctant to take on the responsibility of educating children with SEN and/or disabilities. The first attempts came from private initiatives or charitable organisations and associations, and it was not until 1981 that the legislative framework for special education was first established and later reinforced under Law 1566/1985. At the dawn of the 21st century, inclusion policy was a high priority on the Greek educational agenda, defining for the first time children and young people with ASD as having SEN, while at the same time the first public schools exclusively for children with ASD were established (Law 2817/2000),^5^ mainly in big cities.

The assessment and identification of children with SEN is carried out by multidisciplinary teams at Centres for Multidisciplinary Assessment, Counselling and Support (CEΜΑCS), which operate under the Ministry of Education, as well as by child and adolescent mental health services (CAMHS), which operate under the Ministry of Health. CEMACS have the jurisdiction to suggest the proper educational setting for a child and plan an individual education programme (IEP), implemented by a specialist educator in collaboration with the school advisor. It should be highlighted that the child's parents are encouraged to take part in the process. However, results of previous research suggest that caregivers of children with ASD experience severe stigma, which in turn may affect their decision to access healthcare.^6^ Unfortunately, a number of problems faced by CEMACS as well as CAMHS (e.g. waiting lists, non-permanent staff) make it difficult to fully evaluate and support children in timely way.^7^

To ensure equity and quality, children with high-functioning ASD may attend mainstream school classes full-time alongside their typically developing peers, either without support or with individualised ‘parallel support’ by a person (a co-teacher), who preferably has a specialisation in autism, on a permanent or fixed-term basis (Law 3699/2008).^8^ However, Stefanidis & Strogilos^9^ reported that 37.75% of co-teachers in their study had no qualifications related to special education. Parallel support is provided under the following conditions: (a) ability of the pupil to participate in the classroom curriculum, if supported by a co-teacher; (b) attendance at a rural school, where there is no other special educational setting provided (e.g. on remote islands); (c) a CEMACS report based on a multidisciplinary assessment.

Children with medium or low-functioning ASD may attend a ‘resource class’ within a mainstream school. Resource classes provide two types of educational programme: (a) a specialised programme for all pupils (up to 15 h/week) and (b) an individualised programme with an extended timetable for individuals with more severe SEN. Alternatively, young people with low-functioning ASD may attend either Special Education and Training Units or special schools (Law 3699/2008).^8^

Indicatively, during the school year 2019–2020, 89 597 children with SEN attended general primary and secondary education, 10.7% of whom received parallel support, and 12 086 attended Special Education and Training Units.^10^ In the academic year 2020–2021, 12.6% of the total school population were supported by parallel support and 12 555 children were attending Special Schools. Most of the children in Special Schools were individuals with severe mental health problems, autism or multiple disorders, who were more likely to experience social exclusion due to the severity of their difficulties.^7^

It should be highlighted that according to Law 3699/2008 parents have the right to refuse the recommendations made by CEMACS and register their child in the local mainstream school.^8^ Law 4186/2013 (Article 28, paragraph 18) gave parents the right to hire a ‘special assistant’ to support their child within the mainstream setting, on agreement with the head of the school and the Teachers’ Association of the school.^11^ This allowed a shift from state-funded to privately funded special education provision, as instead of allocating more resources to inclusive education, the state passed on the responsibility to parents. Interestingly, during the 2019–2020 school year, 1222 pupils in primary and secondary education were supported by special assistants hired by their parents.^7^

Last but not least, students with ASD and significant disability certified by a Disability Certification Committee have only recently been admitted to university without participating in the main national exams (such students can constitute up to 5% of the total number of admissions).^12^

## Greek teachers’ perceptions of the educational inclusion of children with ASD

Teachers, as the primary agents of the implementation of the inclusion policy, should have a powerful influence on shaping positive attitudes in that direction. However, quite often they consider themselves insufficiently trained to support children with ASD academically, socially and behaviourally.^13^ Greek teachers were found to be sceptical about the inclusion of children with autism^14^ and many of them tended to believe that their pupils with ASD would be better placed in a special school, learning social skills rather than academic subjects.^15^

## Special education during the COVID-19 pandemic

School closures in March 2020 due to COVID-19 created a new environment with different demands for academic achievement, especially for children with ASD. Communication and socialisation difficulties, and insistence on sameness, specific interests and routines, hindered their ability to respond effectively to the pandemic challenges and adjust to the changes in their daily routine.^16^ The shift to online learning posed a significant challenge to teachers in efficiently adapting the special education curriculum for children with ASD who rely heavily on a structured school environment and often have one-to-one support in the classroom. Tele-education for children with ASD did not follow the individualised instruction and support; they were expected to adapt to distance learning, supported substantially by their parents and caregivers, who, not surprisingly, experienced significant stress.^4,16^ Parents reported outbursts, intense hyperactivity, regression as well sleep and eating problems.^16^ However, in Greek studies about 50% of parents reported that the pandemic-related lockdown had some positive changes in their child's life, including less school-related stress.^17^

Taking into consideration the above-mentioned challenges, in the second period of lockdown, governmental changes in school regulations were differentiated for special schools: these stayed open. Special schools were prioritised for face-to-face teaching, since it was more feasible to maintain social distancing in the classroom, balancing at the same time COVID-19-related risks.

It is worth mentioning that several changes have been made to the way health and social care were provided: psychiatric and psychological assessments were limited, in-patient child and adolescent psychiatry units applied very strict admission criteria, home visits by social workers were reduced and therapeutic programmes and daily group activities were suspended for a long time. The effects of this pandemic and the subsequent measures are expected to be long-lasting for children. In response, the Ministry of Education and Religious Affairs has hired social workers and psychologists to work in mainstream schools, offering specialised and evidence-based guidance to pupils, teachers and parents as well as handling critical issues that arise within the school community.

## Public policy and social justice

Given the commitment that education is a common good, policies must support the inclusion of children and young people with ASD in mainstream education; however, they are not always successfully implemented. Stigma can affect not only the individual with ASD but almost everyone involved. Efforts must be directed towards scaling up the capacity of dedicated teachers with specialised training to tackle stigma, debunk stereotypes, and recognise, reconsider and alter negative beliefs about diversity and human differences.^18^ The main goal is the support of children with disabilities in a flexible school environment. After all, education must be based on inclusion and collective flourishing.

## Data Availability

Data availability is not applicable to this article as no new data were created or analysed in this study.
